# Yeasts in the gastrointestinal tract

**DOI:** 10.1093/femsyr/foag029

**Published:** 2026-06-30

**Authors:** Katherine D Mueller, Soo Chan Lee

**Affiliations:** Department of Immunology and Molecular Microbiology, Texas Tech University Health Sciences Center, Lubbock, TX 79430, United States; Department of Immunology and Molecular Microbiology, Texas Tech University Health Sciences Center, Lubbock, TX 79430, United States

**Keywords:** mycobiome, fungi in GI tract, probiotics, inflammatory bowel diseases, fungal microbiome

## Abstract

The human gastrointestinal (GI) microbiota has come to be recognized as a modulator of health. However, interest in fungi and their function as members of the microbiota has lagged behind interest in bacteria. Despite the lack of historical interest, fungi are prevalent in the human GI tract and have an outsized impact on host immunity. In this review, we aim to examine the associations and potential impact of yeasts on human health outcomes. This review summarizes the associations between yeasts and inflammatory bowel diseases, highlights the predictive service that yeasts may provide in cancer therapy, and explores the possibility of yeasts as therapeutic effectors. There remain significant challenges in data analysis and identifying the relevance of fungal morphology; however, the pathways for clinical translation open to yeasts in the GI tract make these challenges worth overcoming.

## Teaser

This mini review covers how yeasts function as a modulator of human health.

## Introduction

The human gastrointestinal (GI) tract contains complex, interacting microbial communities composed of bacteria, fungi, viruses, protozoa, and archaea. These communities are collectively known as the microbiota; however, this term is commonly used to refer to only the bacterial component of these communities. Thus, the terms mycobiota and mycobiome are used to represent the fungal microbes found in these communities (Ghannoum et al. 2010, Iliev et al. 2012). Analysis of Human Microbiome Project shotgun metagenomic sequencing of stool samples from healthy adults revealed that 0.01% of sequences aligned to fungal genomes, suggesting that fungi represent only a small portion of the microbiota (Nash et al. 2017). This surface-level view may partially explain the preferential focus on bacteria over fungi in studies of the microbiota. However, given that fungal cells can be orders of magnitude larger than bacterial cells, fungal biomass in the GI tract is far greater than genome counts alone would indicate. In fact, the presence of fungi may drive some of the activity of health-relevant bacteria in the GI tract, contributing to an outsized impact of yeasts on health outcomes relative to what their relative abundance may suggest. For example, blooms of the bacterium *Enterococcus faecalis* and *Candida albicans* are often associated with gastrointestinal disease. In a mouse model and *in vitro*, the depletion of environmental glucose by *E. faecalis* leads to increased production of citrate by *C. albicans*, and the resulting increased availability of citrate confers a fitness advantage to *E. faecalis*. Thus, cross-feeding of a bacterium by a yeast allows for the completion of a pro-virulence metabolic cycle in the bacterium (Gause and Johnson 2025).

Humans are inoculated with the beginnings of their mycobiota at birth and reach their adult fungal composition by age 2. At maturity, the dominant taxa include yeasts such as *C. albicans, Saccharomyces cerevisiae*, and *Malassezia restricta* (Gutierrez et al. 2023, Heisel et al. 2025). Importantly, these yeasts are not simply of interest because they happen to be present in the GI tract. The composition of the GI mycobiota has co-evolved with its human hosts. Yeasts such as *Pichia* and *Saccharomyces* are strongly associated with humans, compared to non-human primates, and even taxa that are common across several non-human host species, such as *Candida* and *Kazachstania*, are more prevalent in humans (Van Syoc et al. 2025).

Given the tightly linked evolutionary history of yeasts and their hosts, colonization of the GI tract by yeasts from birth, and the myriad connections between their bacterial neighbors and human health outcomes, it would be natural to suspect that yeasts play an important role in modulating human health. Indeed, this review aims to highlight recent developments in the mycobiome field related to human GI health outcomes. Connections between yeasts and inflammatory bowel diseases will be discussed first, followed by their potential role in modulating cancer development and immunotherapy. Next, the promise of yeasts as probiotic therapeutics will be covered. Finally, we aim to discuss the challenges and limitations of studying the GI mycobiota. Overall, the goal of this review is to emphasize the varied and complex relationships between humans and their lifelong GI yeast companions.

## Inflammatory bowel disease and irritable bowel syndrome

Inflammatory bowel diseases (IBD), such as Crohn’s disease (CD) and ulcerative colitis (UC), are chronic, progressive gastrointestinal diseases. While not yet fully understood, the causes of IBD are multi-factorial and integrated within a complicated web of interactions between human genetics, environment, diet, and the GI microbiota (Torres et al. 2017, Ungaro et al. 2017). Irritable bowel syndrome (IBS) is a non-inflammatory disorder that involves general GI discomfort and irregular stool frequency and consistency (Lacy et al. 2021). Genome-wide association studies have implicated mutations in CARD9, a protein required for inflammatory signaling in response to fungi, as a strong, common genetic risk factor for both CD and UC (Rivas et al. 2011, Jostins et al. 2012). Likewise, Dectin-1 gene variants are associated with increased disease severity in UC, and yeasts, such as *Candida*, can exacerbate disease in genetically susceptible hosts *in vivo* (Iliev et al. 2012). Disease activity in UC is also significantly associated with expression of both Dectin-1 and multiple proinflammatory cytokines, and modulation of Dectin-1 signaling can ameliorate inflammation in mouse models of CD (Takagawa et al. 2018, Azizollah et al. 2024).

The relationship between gastrointestinal fungi and IBD was among the first to garner interest from mycobiota researchers. In these early studies, the links between immune recognition of fungi and IBD were established. Various *Candida* species, including *C. albicans* and *C. tropicalis*, were identified as increased in IBD patients relative to healthy controls. *Saccharomyces* were correlated with health over IBD, and the notion was established that bacteria and fungi may interact in the GI tract such that one or both may exacerbate disease more effectively (Israeli et al. 2005, Iliev et al. 2012, Hoarau et al. 2016, Sokol et al. 2017). *Saccharomyces boulardii* was also shown to prolong disease remission in patients with CD, highlighting the potential for yeast-oriented therapeutic approaches for the management of IBD (Guslandi et al. 2000).

During this time, anti-*S. cerevisiae* antibodies (ASCA), which recognize fungal cell wall components, were identified as potential diagnostic markers for CD and UC, as these antibodies are more prevalent in patient populations with clinically confirmed IBD than in healthy controls (Quinton et al. 1998). ASCA development in serum was also found to predate the diagnosis of CD by a mean of more than 3 years. As a result, ASCA has become a dominant predictive serological marker of CD (Israeli et al. 2005, Paul et al. 2015). Despite the ASCA name implying a starring role by *S. cerevisiae* in the development of IBD, *C. albicans* was later found to stimulate ASCA production (Standaert–Vitse et al. 2006). *Candida albicans* was also identified as more highly abundant in CD patients and their healthy relatives, implicating *C. albicans* as the initial driver of ASCA production and the development of CD (Standaert-Vitse et al. 2009). Other anti-glycan antibodies have also been considered for the characterization of IBD subtypes. Anti-laminarin antibodies bind to laminarin, which is composed of β(1,3)-glycans and β(1,6)-linkages, and anti-chitin antibodies bind to chitin. Adding anti-laminarin and anti-chitin antibodies to ASCA panels can improve their ability to distinguish CD from healthy controls. These markers were also associated with increased disease complications and IBD-related surgery, respectively (Klebl et al. 2010). However, anti-laminarin antibodies alone are not as effective as ASCA alone in distinguishing IBD from healthy controls, and anti-chitin antibodies alone are not discriminatory (Paul et al. 2015).

More recent studies have confirmed and expanded on these results, solidifying the link between gastrointestinal yeasts and IBD and IBS. Genus-level analyses have found that *Candida* and *Malassezia* are significantly more abundant in IBS patients compared to healthy controls (Das et al. 2021). Recent genus-level analyses have also found *Malassezia* and *Naganishia* to characterize the tissue-associated and lumen-associated mycobiota of pediatric CD, respectively (Kim et al. 2025). Surgical resections taken from IBD patients with advanced disease have shown significant increases in *Malassezia* and decreases in *Yarrowia* compared to resections taken from non-IBD patients (Cejudo-Garcés et al. 2025). The following sections will therefore discuss species within the two most commonly referenced yeast groups in the context of inflammatory bowel diseases: *Candida* and *Malassezia*.

Recent analyses, in addition to identifying *Candida* as increased in IBD, have determined that multiple *Candida* species may be involved in disease progression. The most frequently cited *Candida* species in this context is *C. albicans* (Azizollah et al. 2024). However, the closely related *C. dublinensis* has also been correlated with CD and with serological markers of inflammation (Catalán-Serra et al. 2024). High levels of *C. albicans* are also more common in IBD patients who do not respond to treatment with the tumor necrosis factor alpha blocker infliximab, compared to responders (Ventin-Holmberg et al. 2021). The role of *C. albicans* in IBD is likely due to its ability to disrupt the GI epithelial barrier, owed primarily to the production of candidalysin (encoded by *ECE1*). Candidalysin production by the hyphal form of this fungus induces necrotic epithelial damage *in vitro*, allowing for transcellular growth across the epithelial barrier (Allert et al. 2018). Exposure to candidalysin also induces GI epithelial cells to secrete proinflammatory cytokines *in vitro*, suggesting a furthering of the inflammation-to-damage cycle of IBD (Morelli and Queiroz 2025). Further verification using *in vivo* models or humans would be required to fully determine what role this toxin plays in GI inflammation. Treatment of UC with fecal microbiota transplantation (FMT) is most effective for patients with high pre-FMT *Candida* abundances, and the reduction of *C. albicans* post-FMT correlates with decreased disease severity (Leonardi et al. 2020). Additionally, supplementation with *C. tropicalis* exacerbates colitis in mice lacking CX3CR1^+^ mononuclear phagocytes, suggesting a role for this yeast in disease progression in genetically susceptible hosts. This may include human patients carrying the *CX3CR1* T280M allele (Leonardi et al. 2018). Finally, growth of *C. albicans* and *C. glabrata* is inhibited in the presence of the anaerobic GI bacteria, *L. johnsonii* and *B. thetaiotamicron*. In a mouse model of colitis, treatment with *C. glabrata* exacerbated inflammation. However, additional treatment with these bacteria decreased both clinical signs of inflammation and *C. glabrata* load (Charlet et al. 2020). These observations combined suggest that *Candida* reduction in these patient populations may be a promising target for future therapeutics.

*Malassezia* have more recently been identified as potential instigators in IBD. One study found that the *Malassezia* and *Aureobasidium* genera were associated with ileocolonic disease in patients with CD compared to healthy controls. From this, *M. restricta* and *M. globosa* were strongly associated with increased CARD9^S12N^ allele count, suggesting that these yeasts may exacerbate inflammation in genetically susceptible hosts (Limon et al. 2019). Another study identified an increase in *M. globosa* in surgical resections of CD patients compared to non-IBD controls. *M. globosa* and *M. restricta* were also positively correlated with the expression of pro-fibrotic genes (COL1A1 and COL3A1) within the GI tract, indicating promotion of disease progression by these yeasts (Cejudo-Garcés et al. 2025).

Unlike their *Candida* and *Malassezia* neighbors, *Saccharomyces* species appear to play a more regulatory, less pathogenic role in the GI tract. Recent analysis of stool from IBD patients confirmed the decreased abundance of *Saccharomyces* and increased abundance of *Candida* species in CD and UC, particularly in colonic manifestations of CD (Catalán-Serra et al. 2024). *Saccharomyces cerevisiae*, specifically, is decreased in UC compared to healthy controls (Azizollah et al. 2024). Further discussion on *S. cerevisiae*, related yeasts, and their potential in gut health promotion will be covered in a later section on probiotic yeasts.

**Figure 1:**
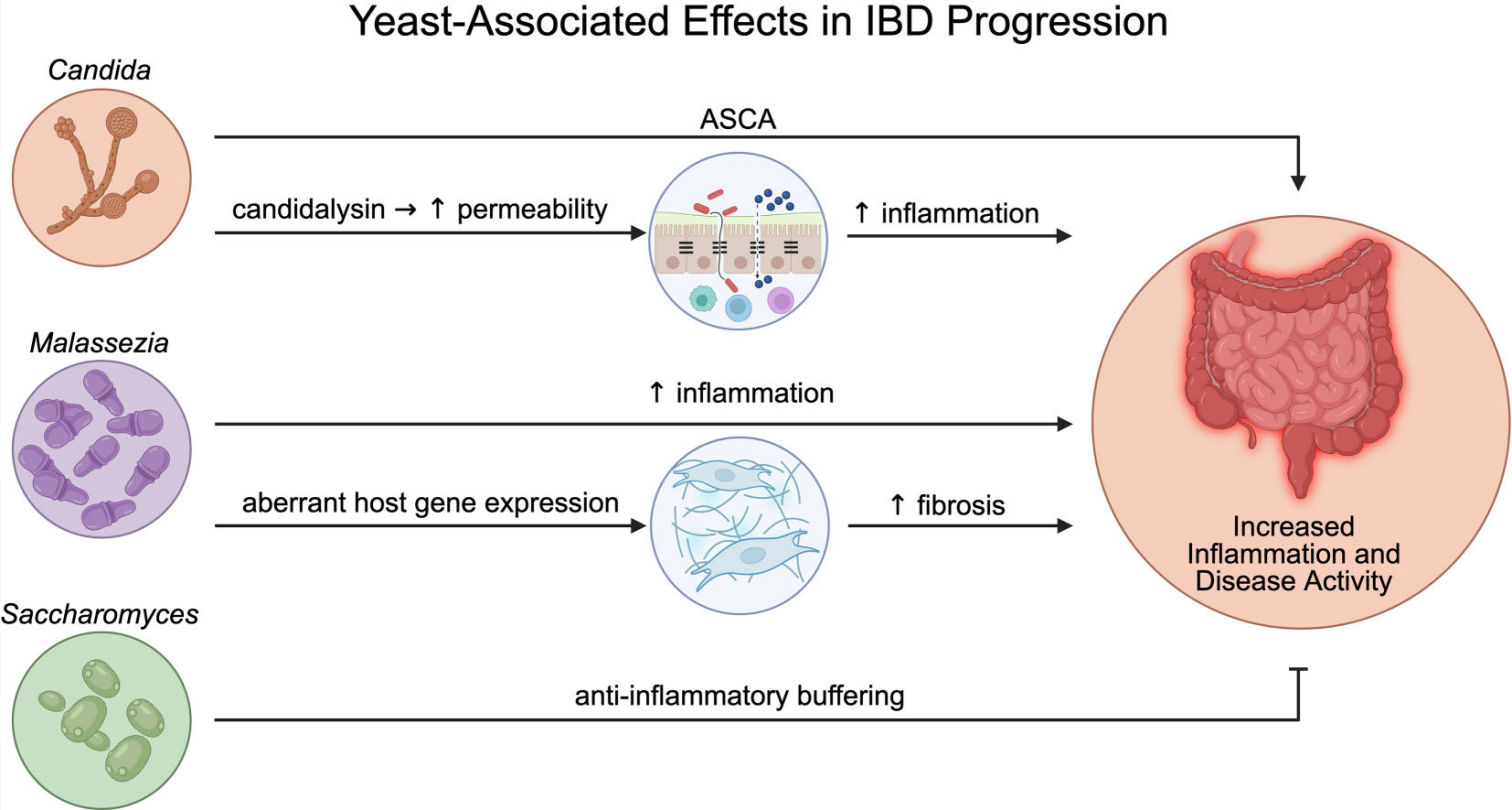
Yeast-associated effect in IBD progression. The yeasts most frequently associated with IBD are *Candida, Malassezia*, and *Saccharomyces. Candida* species promote the production of host ASCA and secrete candidalysin, which contribute to the onset and progression of IBD. *Malassezia* species contribute to the progression of disease by promoting inflammation and fibrosis. *Saccharomyces* may prevent or lessen disease progression through anti-inflammatory activity.

To summarize, the yeasts most frequently associated with IBD are *Candida, Malassezia*, and *Saccharomyces*. Here, we propose a simple model for yeast activity in the progression of inflammatory bowel diseases. Given the role of *Candida* species in ASCA development and the contribution of candidalysin to promoting GI barrier permeability, *Candida* species—particularly *C. albicans*—primarily influence the onset and exacerbation of inflammation in IBD. *Malassezia* species, with their ties to aberrant host gene expression, likely exacerbate disease through both increased inflammation and fibrosis. Finally, *Saccharomyces* likely serves as an anti-inflammatory buffer to prevent or mitigate disease (Fig. 1).

## Cancer

Chronic inflammation, including inflammatory diseases, is a risk factor for multiple cancers due to the contribution of inflammatory cytokines and reactive oxygen species to tumorigenesis and cancer recurrence (Piotrowski et al. 2020). The incidence of gastrointestinal and non-GI cancers is greater in IBD patients than in non-IBD patients. For example, the standardized incidence ratio of colorectal cancer (CRC) in South Korean patients with Crohn’s disease is between 3.7 and 4.7, depending on sex (Jung et al. 2017). Given the link between GI yeasts and inflammation discussed in the previous section, it is likewise worth studying the potential role yeasts play in cancer. In particular, yeasts may serve as non-invasive biomarkers of cancer, enabling earlier disease detection. This section will discuss recent findings concerning the association between yeasts and cancers, both within and outside of the GI tract. Next, the potential of yeasts as predictive markers of progression and cancer therapy will be discussed.

Early studies into the GI mycobiota and cancer established both associative links between yeasts and cancer and the possibility that fungi could serve as diagnostic markers of disease (Yu et al. 2017). In these early studies, yeasts such as *Debaryomyces fabryi* and *Malassezia globosa* were identified as enriched in the GI tracts of patients with CRC. Additionally, *Lipomyces starkeyi* and *S. cerevisiae* were depleted in CRC. Combined with other fungal species, these yeasts could distinguish not only CRC from healthy controls, but also early-stage CRC from late-stage CRC. This diagnostic power was validated in East Asian and European cohorts, demonstrating utility across ethic and dietary variability (Coker et al. 2019). Despite the physical distance and environmental differences between the skin and the GI tract, there is emerging evidence that the reach of GI microbes goes beyond the GI tract and can influence melanoma progression. Recent analysis of stool samples has demonstrated an enrichment of *C. albicans* and *C. dubliniensis* in melanoma patients compared to healthy controls. These patients also display a depletion of *S. cerevisiae, D. hansenii*, and *Sporisorium graminicola* (Szóstak et al. 2024, Szóstak et al. 2025).

Rather than assaying stool, microbial profiling of tumors has implicated fungi in the early onset of pancreatic ductal adenocarcinoma (PDAC). In these cases, *Malassezia* and *Candida* were more abundant in tumors from patients with early-onset PDAC compared to tumors taken from patients with average-onset PDAC (Jayakrishnan et al. 2025). An analysis comparing multiple tumor types demonstrated that *Candida, Saccharomyces, Malassezia*, and *Yarrowia* species are overrepresented in gastrointestinal tumor types, suggesting that tumors in these cancers are seeded by GI fungi (Narunsky-Haziza et al. 2022). Two additional studies profiled fungi within the tumor environment, finding *Malassezia* to be the dominant fungal taxon in PDAC tumors. One of these studies determined that oral administration of *M. globosa* results in enhanced tumor growth and increases the infiltration of ILC2 and T_H_2 cell populations, both required for tumor progression, in a mouse model (Alam et al. 2022). The other found that ablation of the mycobiota was protective against tumor progression in mice, but that restoration of *M. globosa* alone restored the growth of PDAC tumors. This study attributed the action of *M. globosa* on tumor progression to complement activation within the pancreas (Aykut et al. 2019). However, the latter of these studies has since been disputed, based on the high rate of false taxonomic classification involved in the methods used by that study. Reanalysis of this data identified very few fungal reads in tissue samples, calling the reliability of results from such low-biomass samples as tumors into question (Fletcher et al. 2023).

The associations between yeast abundance and the occurrence of cancers have not yet reached a point where causality can be determined. However, monitoring GI yeasts to predict survival, disease progression, and patient responsiveness to therapy holds promise beyond associative studies. For example, patients with high levels of *M. restricta* and *C. albicans* are twice as likely to experience melanoma progression and experience shorter survival times compared to patients with lower levels of these yeasts (Szóstak et al. 2024). Responsiveness to anti–PD-1 immunotherapy is assessed using response evaluation criteria in solid tumors (RECIST). In studies using these criteria, responders to treatment are usually defined as patients with complete or partial responses to therapy, and non-responders are defined as patients with stable or progressing disease (Eisenhauer et al. 2009). A recent study defined favorable-type and unfavorable-type mycobiota that correlated to responsiveness and non-responsiveness to anti–PD-1 immunotherapy across a variety of patient cohorts. In this study, *Kluyveromyces marxianus, S. cerevisiae*, and *Malassezia pachydermatis* were more highly abundant in the favorable-type mycobiota. *Candida tropicalis* and *Candida orthopsilosis* represented the unfavorable-type mycobiota. Importantly, these results were then applied to a mouse model of adenocarcinoma. In this study, mice receiving fecal microbiota transplant of favorable-type stool from human donors experienced significantly reduced tumor growth compared to mice receiving unfavorable-type stool (Hu et al. 2025). This study exemplifies the potential for manipulating yeasts in the GI tract to improve therapeutic efficacy.

## The potential of probiotic yeasts

Thus far, yeasts in the gastrointestinal tract have primarily been discussed as associated with health outcomes, often when overrepresented in diseased states relative to healthy controls. Understanding the pathogenic potential of these yeasts and engineering solutions to inhibit their disease-potentiating effects is, of course, an important goal. However, focusing only on those disease-associated fungi would deny the potential of other yeasts in supporting gastrointestinal health. This section will discuss the history of, and recent advances in, scientific interest in yeasts as therapeutic agents. As the yeast group with the most historical interest, *Saccharomyces* will be covered first, followed by non-*Saccharomyces* yeasts. Finally, the potential of yeasts as therapeutic delivery vessels, rather than as primary actors, will be introduced.

As discussed in the previous section, *Saccharomyces* species were recognized as health-related yeasts during the earliest assessments of fungi in IBD. Given their seemingly contradictory associations with ASCA, additional studies soon began to investigate the role that these yeasts play in the GI tract and in inflammation. *Saccharomyces cerevisiae var. boulardii*, often published and referred to in common media as *Saccharomyces boulardii*, has thus far garnered the most interest as a fungal probiotic. A recent pangenomic analysis of several *S. boulardii* and *S. cerevisiae* strains has demonstrated that *S. boulardii* strains have smaller genomes and fewer singleton genes than typical laboratory strains (Duffey et al. 2026). Functional and metabolic analyses revealed that *S. boulardii* strains contain different numbers of carbohydrate-active enzyme families and unique sequence variation in central carbon and tryptophan metabolism genes. These findings were reflected *in vitro—S. boulardii* strains exhibit higher survival in gastric and intestinal fluids, produce more acetate and succinate, and reduce inflammatory cytokine signaling in intestinal cell lines compared to laboratory strains. An early pilot trial determined that *S. boulardii* could reduce the clinical index score in UC patients for whom steroid therapy was unsuitable (Guslandi et al. 2003). Additional studies found that treatment with *S. boulardii* can inhibit the exacerbating effect of *C. albicans* colonization on induced colitis in mice and that stimulation of dendritic cells with *S. boulardii* increases anti-inflammatory IL-10 *in vitro* (Jawhara et al. 2012, Sokol et al. 2017). Furthermore, *S. boulardii* prevents systemic *Salmonella typhimurium* infection by modulating IL-10 and increasing the rate of bacterial transit through the mouse GI tract (Pontier-Bres et al. 2014). Daily administration of *S. cerevisiae* to mice also prevents the induction of colitis by adherent-invasive *Escherichia coli*, further solidifying the potential of these yeasts as therapeutics for IBD (Sivignon et al. 2015). Most recently, a clinical trial demonstrated that twice-daily administration of *S. boulardii* effectively improved diarrhea-dominant pediatric IBS by normalizing bowel movements, increasing stool hardness, increasing IL-10, and decreasing multiple inflammatory cytokines (Jin et al. 2025).

Though interest in yeasts as therapeutic agents began with *Saccharomyces*, additional yeast taxa have garnered interest in recent years. *Kazachstania pintolopessi* mediates intestinal healing, and a peptide fragment of Ygp1 from this yeast is sufficient to treat mouse models of colitis and chemotherapy-induced intestinal injury. *Kazachstania pintolopessi* is a colonizer of the mouse GI tract but is not typically found in humans. However, the Ygp1 peptide fragment does support the differentiation of human intestinal organoids. Additionally, this peptide fragment can be produced by *E. coli* and does not induce toxicity in mice. While human clinical trials involving the Ygp1 studies have not occurred, the human *in vitro* data, mouse model efficacy, and mouse safety profile together do suggest translational value for the treatment of GI diseases (Gao et al. 2026). Meta-analysis of stool samples from Chinese IBD patients revealed that *Clavispora lusitaniae* (formerly *Candida lusitaniae*) is consistently depleted across cohorts. Follow-up *in vitro* and *in vivo* further revealed that treatment with *C. lusitaniae* alleviates clinical symptoms of colitis and promotes epithelial barrier repair. These effects are due to the production of indole-3-ethanol (IEt) by *C. lusitaniae*, which activates intestinal repair pathways in an AHR-dependent manner. The anti-colitis effect of this fungus can be modulated by increasing IEt production; thus, *C. lusitaniae* engineered to produce enhanced levels of IEt has potential as an auxiliary treatment for IBD (Wu et al. 2025).

Beyond any inherent benefits that yeasts and their metabolites may provide, there is also potential for yeasts as therapeutic delivery systems. *Saccharomyces boulardii* is currently being developed to secrete a rotavirus vaccine candidate fusion protein. Despite the end product demonstrating poor immunogenicity in mice, *S. boulardii* secreted a stable, nontoxic predicted antigen, demonstrating the potential of this yeast as a GI-targeted vaccine delivery vehicle (Farhani et al. 2026). Endolysins are phage-derived antimicrobials that are highly specific to their target bacterial taxa. Using a CRISPR-Cas9 system, *S. cerevisiae* and *S. boulardii* have been engineered to secrete Ply511; an endolysin which is active against all serovars of *Listeria*. Secretion of Ply511 inhibits *Listeria* without disrupting other common GI bacteria in *in vitro* models. Applied to a clinical setting, further development of these strains would allow for clearance of *Listeria* infection from the GI tract in a targeted manner. More broadly, with some optimization of these *Saccharomyces* strains for better expression under anaerobic conditions, this system could serve as a platform for targeted interventions in bacterial infections of the GI tract (Moreno et al. 2026). Aside from *Saccharomyces*, supplementation of feed with *Yarrowia lipolytica* decreases coliform bacterial load in piglets (Czech et al. 2018). *Yarrowia lipolytica* is an efficient producer of heterologous proteins, and engineered strains are already used to produce therapeutic proteins outside of the GI tract. The recent development of fluorescently labelled strains has revealed that orally administered *Y. lipolytica* reliably survives conditions within the mouse GI tract but does not permanently colonize the host (Madzak et al. 2023). Thus, this yeast may be appropriate for the as-needed production of therapeutic agents within the GI tract. The relevance or benefit of these observations to the human gastrointestinal tract is currently unclear. However, given the previously established associations between *Yarrowia* species and IBD and cancer in humans, these yeasts may serve as yet another delivery vehicle for therapeutic interventions for GI disorders.

## Challenges

Many fungi exhibit dimorphic growth, adopting either yeast or filamentous forms that are associated with distinct transcriptional profiles. Thus, distinguishing between these forms represents a challenge unique to the mycobiota. Currently, most studies utilize ITS amplicon sequencing to identify components of the mycobiota. However, these sequences only reveal which fungi are present, not which form they take. For example, the black mold, *Mucor*, can switch between hyphal and yeast modes of growth (Vellanki et al. 2020). *Mucor* species have been associated with inflammation in the gastrointestinal tract and may alter the bacterial microbiota of healthy individuals towards a more pro-inflammatory composition (Mueller et al. 2019, Huang et al. 2026). But are these *Mucor* species acting as yeasts or hyphae within the GI tract? ITS amplicon sequencing cannot tell us this, and thus, it is unknown whether this dichotomy is relevant to health outcomes. In fact, this distinction between yeast and hyphal growth may be key to understanding the relationship between fungi and host health outcomes.

Many early studies of the mycobiota identified taxa at the genus level or higher. Given that fungal species can vary widely within even one genus—*Aspergillus*, for example, is composed of hundreds of species—it is critical that future studies focus more heavily on species- and perhaps even strain-level differences (Houbraken et al. 2020, Hu et al. 2025). Early mycobiota studies also used OTU picking, which clusters sequences based on a fixed similarity threshold. By combining similar sequences, OTU picking reduces the rate at which sequencing errors are misinterpreted as meaningful biological variation, but resolution is limited by the similarity threshold (Caporaso et al. 2010). This method is increasingly recognized as inappropriate for microbial taxa identification and quantification compared to DADA2. The DADA2 method infers sample sequences exactly, empowering DADA2 to distinguish between sequences that differ by a single nucleotide. This allows for much greater resolution and differentiation between closely related strains (Callahan et al. 2016). The discrepancies between these methods have resulted in some disagreement as to the importance of *Malassezia* in the development of pancreatic cancer (Aykut et al. 2019, Fletcher et al. 2023). Thus, it may then be useful to revisit or verify early works that utilized OTU picking before basing future studies on their results. Additionally, more up-to-date methods of identifying taxa, such as DADA2, should be adopted for future studies. This would bring the mycobiome field more in line with that of the bacterial microbiota, in which DADA2 has been adopted as the standard for 16S rRNA amplicon sequencing analysis.

The methods used to sequence microbial DNA samples pose another challenge for studies of the mycobiota. Currently, the two most common methods involve either amplicon sequencing (18S, ITS1, or ITS2) or shotgun metagenomic sequencing. Amplicon sequencing limits the extractable information about a microbe to taxonomic information—investigators can determine the genus and, possibly, species of a fungal taxon, but are offered no information about genome-wide variability between different strains within those taxa. Meanwhile, shotgun metagenomic sequencing confers the potential to explore intra-species variability. This type of analysis is becoming more common for studies of the bacterial microbiota but is often limited in resolution for fungal samples (Pasolli et al. 2019, Avershina et al. 2025). Additionally, the application of these two approaches may influence the number and type of observed species. For example, amplicon sequencing has been shown to identify more *Candida* species, and shotgun metagenomic sequencing has been shown to identify more *Aspergillus* species in the same dataset (Heisel et al. 2025). This highlights yet another challenge that can interfere with our view into gastrointestinal yeast composition.

Finally, the question of whether these potentially health-relevant fungi are true residents of the GI tract vs transient visitors has yet to be settled. Yeasts are involved in the production of countless food products, including cheeses, breads, wines, and fermented foods around the world. Indeed, one of the early mycobiome-oriented studies noted that the abundance of *C. albicans* was correlated with recent carbohydrate consumption (Hoffmann et al. 2013). The GI tract may also be transiently exposed to skin-associated yeasts, such as *Malassezia*, via an oral route. However, a recent study has provided evidence that IBD-associated *Malassezia* are likely resident members of the GI mycobiota (Cho et al. 2025). In this study, *M. globosa* isolates from patients with ulcerative colitis were found to be less oxygen-tolerant and to exhibit enhanced growth under low-oxygen conditions relative to skin-derived isolates. This suggests that *M. globosa* strains in the GI tract have adapted to GI conditions and thus are likely not simply passing through the GI tract after consumption or migration from host skin. The mating transcriptional network of *C. albicans* has also been shown to have been adapted for commensalism in a mouse model (Witchley et al. 2021). Further investigation into GI-adaptations by additional yeasts, such as those mentioned in this review, would support this resident-mycobiota position.

## Outlook

Despite these significant challenges, the potential upsides of harnessing and manipulating yeasts in the gastrointestinal tract are enormous. This review covered the association between multiple yeasts, inflammatory bowel disease, and cancer progression. These yeasts represent targets for future therapeutics, whereby the reduction of overrepresented fungi may cut off sources of inflammation and oncogenesis. These fungi may also be used as diagnostic markers and predictors of therapeutic outcomes, thereby assisting physicians in their determinations of which therapeutic avenues to use on a patient-by-patient basis. Indeed, disease prediction models that consider both the bacterial and fungal microbiota may be more accurate than those that incorporate only one or the other (Lin et al. 2022). This review also covered yeasts that hold the potential to improve human health outcomes. These yeasts may be used as probiotics that inhibit inflammatory cytokine signaling or prevent the colonization of the GI tract by pathogenic microbes. Alternatively, yeasts can be genetically engineered to produce peptides and metabolites they would not naturally synthesize, allowing them to function as miniature manufacturing hubs for therapeutics and vaccines.

**Figure 2:**
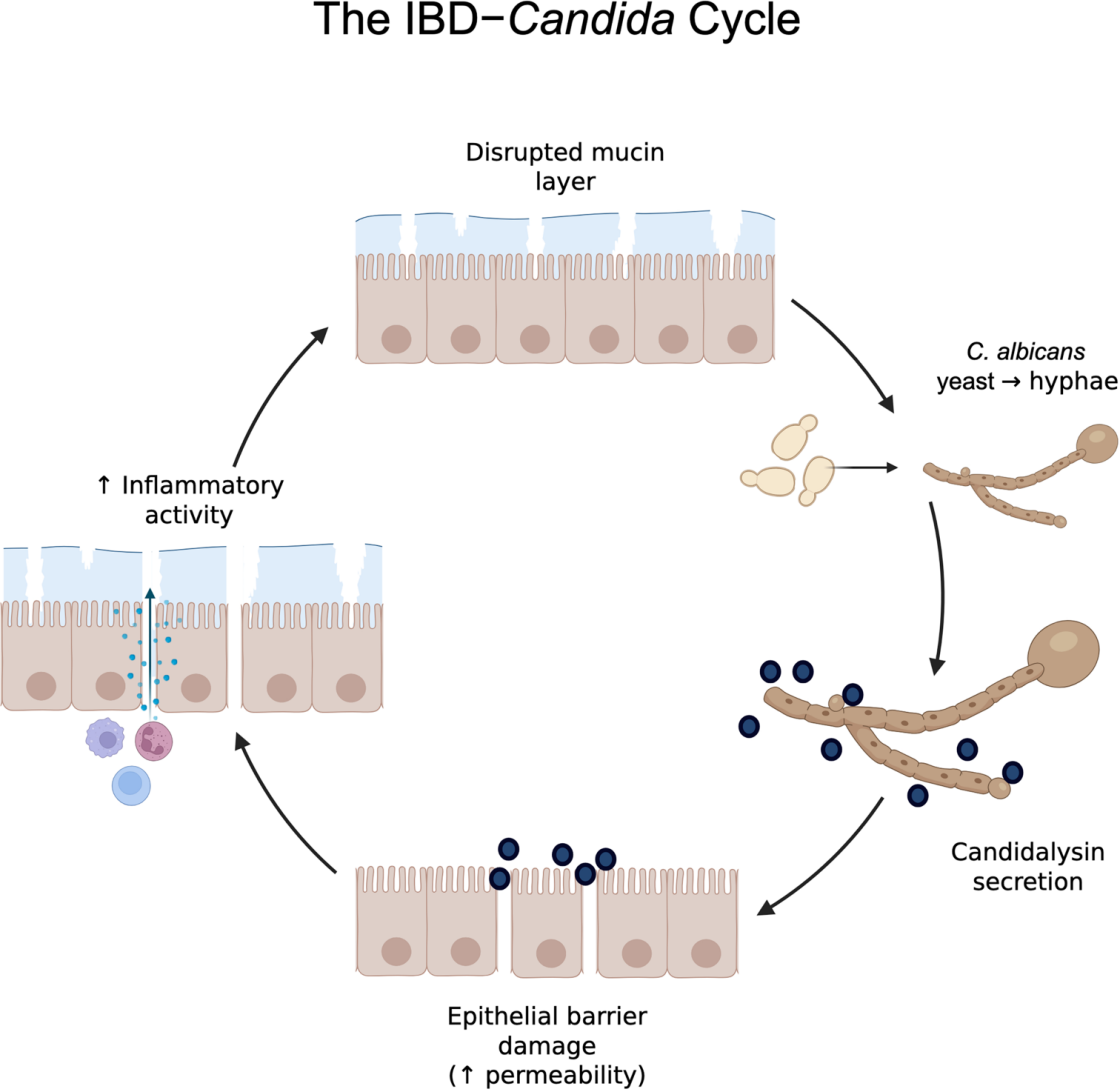
The IBD–*Candida* cycle. Disruption of the gastrointestinal mucin layer may allow *C. albicans* to more easily transition to the hyphal form. *Candida albicans* then produces candidalysin, which disrupts the epithelial barrier. This disruption then contributes to increased immune activity and inflammation in IBD.

As mentioned earlier, despite posing a significant challenge to overcome, the ability to distinguish yeast from hyphal growth may be key to our understanding of fungi and host health outcomes. Interestingly, GI mucins, including the intestine-predominant MUC2, inhibit the ability of *C. albicans* to undergo the yeast-to-hyphae transition. Exposure to mucin O-glycans downregulates over 200 *C. albicans* genes, and 20% are associated with filamentous growth and virulence factors. This suppression of hyphal growth occurs in an Nrn1-dependent manner, is dose-dependent, and does not affect yeast-form growth (Takagi et al. 2022). This leads us to propose an interesting concept for how *C. albicans* promotes inflammation in inflammatory bowel diseases and how future therapeutics may target *C. albicans* activity. In this model, which we will call the IBD–*Candida* cycle, mucin production under homeostatic, or healthy, conditions inhibits the yeast-to-hyphae transition of *C. albicans* in the GI tract. When mucin production becomes disrupted, as may be the case in IBD, *C. albicans* is better able to transition to the hyphal form. As the production of candidalysin is specific to hyphae, this transition may then lead to increased candidalysin production (Liang et al. 2024). Finally, increased candidalysin at the GI barrier disrupts that barrier, contributing to immune infiltration and additional inflammation that exacerbates IBD symptoms (Fig. 2). Under this model, preventing or controlling the yeast-to-hyphae transition in gastrointestinal *C. albicans* could be a prime target to cut off one source of inflammation in IBD.
